# Assessing the Determinants of the Wish to Die among the Elderly Population in Ghana

**DOI:** 10.3390/geriatrics6010032

**Published:** 2021-03-23

**Authors:** Sally Sonia Simmons, Valeria Maiolo, Bright Opoku Ahinkorah, John Elvis Hagan, Abdul-Aziz Seidu, Thomas Schack

**Affiliations:** 1 Department of Social Policy, London School of Economics and Political Science, London WC2A 2AE, UK; ssimmons@edu.hse.ru; 2 Institute of Demography, National Research University-Higher School of Economics, 109028 Moscow, Russia; 3 Department of Legal, Historical, Economic and Social Science, Magna Græcia University of Catanzaro, Viale Europa, 88100 Catanzaro, Italy; valeriale.maiolo@gmail.com; 4 The Australian Centre for Public and Population Health Research (ACPPHR), Faculty of Health, University of Technology Sydney, Sydney, NSW 2007, Australia; brightahinkorah@gmail.com; 5 Department of Health, Physical Education & Recreation, College of Education Studies, University of Cape Coast, Cape Coast CC123, Ghana; 6 Neurocognition and Action Research Group—Biomechanics, Faculty of Psychology and Sports Science/CITEC, Bielefeld University, Postfach 10 10 31, 33501 Bielefeld, Germany; thomas.schack@uni-bielefeld.de; 7 Department of Population and Health, University of Cape Coast, Cape Coast CC123, Ghana; abdul-aziz.seidu@stu.ucc.edu.gh; 8 College of Public Health, Medical and Veterinary Sciences, James Cook University, Townsville, QLD 4811, Australia

**Keywords:** elderly, Ghana, public health, risk factors, suicide, wish to die

## Abstract

Background: A wish to die is common in elderly people. Concerns about death wishes among the elderly have risen in Ghana, where the ageing transition is comparable to other low-and middle-income countries. However, nationally representative research on death wishes in the elderly in the country is not readily available. Our study aimed to assess the determinants of the wish to die among the elderly in Ghana. Methods: We analysed data from the World Health Organisation Global Ageing and Adult Health Survey, Wave 1 (2007–2008) for Ghana. Data on the wish to die, socio-demographic profiles, health factors and substance abuse were retrieved from 2147 respondents aged 65 and above. Ages of respondents were categorised as 65–74 years; 75–84 years; 85+ to reflect the main stages of ageing. Logistic regression models were fitted to assess the association between these factors and the wish to die. Results: Age, sex, place of residence, education, body mass index, hypertension, stroke, alcohol consumption, tobacco use, income, diabetes, visual impairment, hopelessness and depression had statistically significant associations with a wish to die. Older age cohorts (75–84 and 85+) were more likely to have the wish to die (AOR = 1.05, CI = 1.02–1.16; AOR = 1.48, CI = 1.22–1.94), compared to younger age cohorts (65–74 years). Persons who felt hopeless had higher odds (AOR = 2.15, CI = 2.11–2.20) of experiencing the wish to die as compared to those who were hopeful. Conclusions: In view of the relationship between socio-demographic (i.e., age, sex, education and employment), hopelessness, anthropometric (body mass index), other health factors and the wish to die among the elderly in Ghana, specific biopsychosocial health promotion programmes, including timely identification of persons at risk, for appropriate intervention (e.g., psychotherapy, interpersonal support, alcohol-tobacco cessation therapy, clinical help) to promote their wish for a longer life is needed.

## 1. Introduction 

The wish to die is a multi-dimensional concept that builds on three core elements; motivations, social interactions and intentions [1]. Elsewhere, the wish to die refers to suicidal ideation [2,3], which specifically defines the will to die as thoughts of taking one’s own life. The will to die, considering hastening death with thoughts of speeding up death at a later time, and not considering hastening death but with a desire to die are variants of intentions related to the wish to die. Contextual analysis of wish to die intentions suggests that the desire to die antedates thoughts of hastening death and killing one’s self. In this regard, the desire to die is the initial intention because persons who wish to die first crave death before they think about inducing death [1]. Hence, in contrast to existing scholarship [2,3], which equates the wish to die with the will to die, the wish to die is defined here as a desire to die without hastening death [1]. 

Globally, about 10–20% of all elderly people express a wish to die [4] and this estimate is expected to rise as the number of persons aged 65 and above continues to increase [5]. The elderly are at a higher risk of completed suicide following a wish to die than any other group [2,6,7,8] and 73% of all completed suicide occur in developing countries, even though they are under-reported [9]. From 2000–2016, suicide rates in sub-Saharan Africa (SSA) decreased from 8.5 to 7.5 per 100,000 population. Within this period, SSA countries like Ghana and Cote D’Ivoire recorded an increase in suicide from 4.0 to 5.4 per 100,000 people (4.0% to 8.7% among males and 1.9% to 2.1% among females), and 9.5 to 14.5 per 100,000 people (13.7% to 20.6% among males and 5.0% to 8.3% among females), respectively [10]. Also, the desire to die has been found to be associated with mental disorders and other risk factors [2,4,11,12]. However, in Ghana, where mental disorders represent 9% of the disease burden [13] and the number of individuals aged 60 years and over increased from 215,258 in 1960 to 1,643,978 in 2010 (a 770% rise in the elderly population in terms of absolute numbers) [14], information on the association between the wish to die, mental disorders and other risk factors in the old-aged is limited. 

The interpersonal suicide theory includes a complex range of biopsychosocial (immutable, e.g., age, sex, and modifiable, e.g., disease, income, social support, depression, disability) factors relevant to the understanding of the desire to die in Ghana [3,11,15,16,17,18]. Older males and females who face economic difficulties depend on others for survival, lose self-worth and lack societal recognition [19,20]. About 15% of the older Ghanaian population who wished they were dead and committed suicide were indebted and had other unmet financial needs [21,22,23]. Quarshie [17] has added that older people who face economic difficulties have depressive thoughts and poorer mental health. The elderly, especially males, develop different coping strategies such as smoking marijuana and tobacco use, alcohol consumption, intake of sedatives and other substances [20,24]. These coping strategies are stigmatised, receive negative publicity and increase disease susceptibility [24,25]. The stigma and negative publicity distort the victims’ social ties, facilitate loneliness and further intensify their desire to die [22]. Most ethnic and religious groups consider the desire to die as sinful [26,27]. For example, Akans, the most populous ethnic group in Ghana, maintain that the desire to die and suicide attempts are an offense against their supreme deity, ancestral spirits and the living [21,22]. 

Ghanaians are becoming biologically older and the wish to die increases as a function of age. Other social factors, such as social networks where older people connect with friends and family, provide a further understanding of death wish intentions. Also, financial need and reduced opportunities for social interaction may play a role in whether or not the elderly have the desire to die. Another important factor is how the elderly deal with the complexities of mental and emotional challenges and the biological and social demands of ageing. Hence, our study assessed the determinants of the wish to die from a biopsychosocial perspective using self-reported factors from a large-scale study of Ghanaians. 

## 2. Materials and Methods

### 2.1. Data

The data for the study were acquired from the World Health Organisation (WHO) Global Ageing and Adult Health, (SAGE) Wave 1 for Ghana (2007–2008). The SAGE Wave 1 study is a longitudinal study of persons aged 50 years and older, together with a comparable sample of people aged 18–49 years, from nationally representative samples in six countries, including Ghana. A total of 5573 Ghanaians 18 years and above were recruited using multistage cluster sampling techniques [28]. Of the 5573 surveyed, 2147 were eligible for the present study because they were 65 years or older at the time of the study. A detailed description of the SAGE Wave 1 study methods and materials, including the data and data collection procedures, are provided on the project website.

### 2.2. Variables

The wish to die among the elderly was the outcome variable for the study. It was defined as the proportion of people aged 65 years and above who either had “yes” or “no” as responses to “Did you wish you were dead?” Twenty-seven variables were selected as predictor variables. These factors included demographic variables (age, sex, marital status, education, religion, ethnicity, place of residence), disability (hearing, visual, physical), substance abuse (tobacco smoking, alcohol consumption), socioeconomic variables (employment, wealth quintile), hopelessness, social connectedness (attending religious activities, having friends over and social meetings outside home), and health factors (psychological factors were represented by depression, physical activity, comorbidities including diabetes, hypertension, arthritis, stroke, asthma, injury, and the body mass index (BMI)). The selection of these variables was per existing literature and to achieve numerically stable and adaptable models [29]. While age and BMI were numeric variables, sex, diabetes, hypertension, arthritis, stroke, asthma, hearing impairment, visual impairment, physical challenges, injury, tobacco use, alcohol consumption, place of residence, hopelessness, and employment were dichotomous variables. Level of education, religion, ethnicity, wealth quintile, attending religious activities, having friends over, social meetings outside home, and marital status were polychotomous. Age and BMI were transformed to categorical variables to reflect the main stages of ageing (65–74: young old, 75–84: old Akan’s, 85+: oldest old) [30], and BMI (<18.5: underweight; 18.5–24.9: normal; 25.0–29.9: overweight; ≥30.0: obese) [31,32,33]. Marital status was transformed from a polychotomous to a dummy variable, with married and not married as the new categories. The not married category included all Ghanaians who were never married, divorced, separated or widowed. Religion was transformed into a six-category variable, namely, no religion, Christianity, Islam, African traditional, other and refused to answer. Level of education was transformed from a six to four-category variable, that is, no education, primary, secondary and tertiary. Ethnicity was categorised as Akan, Ewe, Ga-Adangbe, Gruma, Grusi, Guan, Mande-Busanga, Mole-Dagbani, others. Attending religious activities, having friends over and social meetings outside home were transformed to dummy variables with the following categories: low (1 = never, 2 = 1 to 2× per year, 3 = 1 to 2× per month) and high (4 = 1 to 2× per week, and 5 = daily). The frequency of missing data was computed to identify the pattern of missingness [34]. Although 20% of the data were missing, missingness was conditional on another variable (missing at random) [35]. Therefore, missing values for the variables were imputed using the chained equations imputations, given the type of variables under study [36]. Following this, persons 65 years and above were included in the study. The sample for the study became 2147.

### 2.3. Data Analyses

Summary statistics of the variables of interest were estimated. The outcomes of the summary statistics were presented as percentage distributions to provide a clearer understanding of the distribution of these factors in Ghana. A chi-square analysis was done to identify factors associated with the wish to die among the elderly in Ghana. The least promising variables were filtered out, and twenty five factors that showed a statistically significant (*p* < 0.05) association (hopelessness, attending religious activities, having friends over and social meetings outside the home, age, sex, residence, marital status, education, ethnicity, religion, physically challenged, tobacco use, alcohol consumption, employment, depression, physically active, diabetes, hypertension, arthritis, stroke, visual impairment, BMI, injury, and wealth quintile) were included in the subsequent multivariate analysis. The test of independence was introduced to improve the performance of the primary model and to eliminate modelling issues such as over-fitting. A logistic regression model was fitted to ascertain how predictors contributed to the prediction of the response variable [37]. The results from the models were presented as adjusted odds ratios (AOR). For all estimations, the reference value for the response variable the wish to die, was coded “1”, and no wish to die, was coded as a “0”. For a given factor, each element selected as a reference was informed by the existing literature. Statistical significance was set at a 95% confidence level and complemented by a two-sided probability set at *p* < 0.05. The percentage of variation in the response variable (death wish) explained by predictors in the model was estimated. All analyses were weighted and performed using survey, dplyr and extraoperator packages in R statistical software version 4.04 [38,39,40].

## 3. Results

### 3.1. Descriptive Statistics and Association between Predictor Variables and the Wish to Die

The distribution of the wish to dies and the relationship between the wish to die and classifiers such as age, sex, BMI, diabetes, hopelessness, and impairment are presented in Table 1. Regarding the prevalence of the wish to die in the country, it was found that almost half (1000, that is ~47%) of the elderly population wished they were dead. More females (54.4%) as compared to males (45.6%), rural residents (61.3%) compared to urban residents (38.7%), Christians (62.0%), Akans (41.5%), persons aged 65–74 (58.9%), persons who felt hopeless (86.5%), and alcohol consumers (57.9%) wished they were dead. The relationship between these classifiers and the wish to die differed. Except for asthma ($\chi^{2}=0.24,\;p>0.1)$ and hearing impairment ($\chi^{2}=0.43,\;p>0.1)$, all variables had a statistically significant relationship with the wish to die. 

### 3.2. Regression Analyses on the Predictors of the Wish to Die

Table 2 depicts the results of the predictors of the wish to die among the elderly population in Ghana. After adjusting for the variables, findings showed that older age cohorts (i.e., 75 and above years) unlike younger elderly age cohorts (65–74 years) had higher odds of having the wish to die. Females were less likely (AOR = 0.48, CI = 0.30–0.95) to wish for death than males. Elderly who are not married were more likely (AOR = 1.63, CI = 1.49–1.81) to wish they were dead as compared to those who were married. Elderly individuals with rural residential status were less likely to have the wish to die compared to persons who were urban dwellers. Elderly persons who had hypertension and stroke were more likely (AOR = 1.49, CI = 1.36–1.66; AOR = 1.28, CI = 1.10–1.86) to wish they were dead. The elderly who felt hopeless were more likely (AOR = 2.15, CI = 2.11–2.20) to wish they were dead as compared to those who were hopeful. Persons who were obese and underweight were more likely to have the wish to die than those who had normal weight. About 48% of the variation in the response variable was explained by the predictors in the model (R^2^ = 48%). 

## 4. Discussion

The current study assessed the association between a range of factors and the wish to die among the elderly population in Ghana. Findings showed that socio-demographics, chronic health conditions, hopelessness and social connectedness were significantly associated with an increased wish to die. Of the 2147 elderly Ghanaians sampled, about a half (~47%) had experienced a wish to die. This prevalence rate is higher than the 10% to 20% reported in Italy [41] and the Netherlands [42], and the ~3% reported in 16 African countries [43], which might, in part, be due to differences in the meaning of the wish to die across different religious, cultural and economic settings as well as increased geriatric mental healthcare challenges [3,12,13,16,17,22,26].

Ghanaians who were most likely to express the wish to die felt hopeless, were older (75 years and above), unmarried, depressed, unemployed, and living with stroke and hypertension. Conversely, females, persons with some level of education, without disability (i.e., visual and physical), who do not engage in substance abuse (e.g., tobacco smoking, alcohol consumption), not depressed, diabetic, or injured were more likely not to wish for death. These findings correspond with research indicating that chronic health conditions, hopelessness, social isolation, disability, and financial difficulties result in increased susceptibility to the wish to die among the elderly [1,4,17,22,44].

Consistent with previous studies [2,45,46] linkages between the socio-demographics (e.g., age, sex, marital status) and the wish to die were established in the current study. It was revealed that males were more likely to have the wish to die than females, a finding consistent with international trends where three to four more men wish to die and die by suicide than women [43]. The substantial variation in the wish to die between men and women, mirrors their roles in society [17]. Being in a marital union was associated with a decreased wish to die. Studies [47,48] have shown that the elderly who are not married, generally report poorer health, show death ideations and have a greater mortality risk as compared to those who are married. Corna et al. [2] explained that marriage provides social and emotional stability, which is not obtained among those who are divorced, separated, single or widowed. These benefits offer the best protection against the wish to die and potential suicide attempts because of its ability to enhance social and community integration, and therefore, decrease social isolation [47,49,50]. The implication is that promoting older couples’ togetherness and engagement in community actions (i.e., beyond simple community membership), such as voluntary physical activities or interacting with other people, may reduce the risk of having death thoughts.

Individuals who had wealth and had some level of formal education were less likely to wish they were dead. Previous studies have shown that high socio-economic status is a protective index against the wish to die among the aged [51]. Education has been considered one of the most essential socio-economic factors since it forms the basis for future occupational opportunities and income. Education provides knowledge and life skills that allow educated people to gain more ready access to information and resources that promote their health [52]. Similarly, it increases human capital, boosts productivity, augments lifetime earnings, and improves an individual’s social connection, which mitigate possible perilous psychological thoughts that can induce the wish to die [53]. By creating employment and income opportunities, education provides means for seeking health care, better nutrition, housing and recreation, which can protect the aged from developing a wish to die [54]. On the contrary, financial difficulties, hopelessness and reduced societal connectedness may lead to severe feelings of dejection or loneliness, and the subsequent wish to die [1,3,11]. These challenges provide further evidence for the importance of social support for the elderly years of life. 

Medical health conditions like diabetes, hypertension, stroke and vision impairment have an influence on the wish to die [11,55]. Illnesses, in particular, generate stress in life, increase the burden on caregivers, cause family discord and drain financial resources. These externalities reduce the victim’s existing wish to live, and therefore, they may wish to die [56]. The comorbidities of physical challenges, vision impairment, injuries or chronic health conditions include depression and other psychological factors. Accordingly, depression may intercede between physical challenges, vision impairment, injuries or chronic health conditions and the likelihood of having the wish to die. 

Alcohol consumption and tobacco use were also associated with the wish to die among the elderly Ghanaian population. Many studies have found a connection between substance use and the wish for death [57,58]. Regular consumption of alcohol and excessive use of tobacco leads to chronic health problems, hypertension, stroke, liver disease, and cancer [59]. These health conditions have detrimental effects on patients’ mental health (i.e., depression, cognitive impairment) [4,60,61], which might increase an individual’s susceptibility to developing the wish to die [61]. Also, as the elderly degenerate because of these chronic health conditions, their capacity to alleviate their health challenges declines, so may wish to die as a plausible solution to end their struggles [1,16,62].

### 4.1. Practical Implications

The current findings have important clinical and social welfare implications for geriatric mental healthcare and non-communicable disease management. Information provided on the biopsychosocial correlates of the wish to die would help in the early detection of individuals at higher risk of potential suicide attempts through death ideations, and ensure appropriate interventions. Health and social welfare institutions could collaborate to design biopsychosocial interventions (e.g., psychotherapy, alcohol and/or tobacco cessation therapy, interpersonal support, clinical help) that promote positive adaptation in old age. Undertaking a longitudinal study on the themes and possible inclusion of variables (e.g., degree of dependence, diagnosis, length of survival since diagnosis, and wish to die attributes; e.g., duration, severity, or variability) not captured in the current study may assist with illuminating the association between wish to die and biopsychosocial health in Ghana.

### 4.2. Strength and Limitations

The present study adds to existing literature by showing gender variations in the determinants of the wish to die among older people. The use of the population-based nationwide survey makes the findings generalisable to other homogenous geographical settings in Africa. There are, however, limitations that should be acknowledged. First, wish to die was measured as a dichotomous variable and does not account for the other attributes of the wish to die, including the duration, severity, or variability of that intention. Second, since the data used for the study was collected more than a decade ago and about one-fifth of the data was missing, the number and types of self-reported cases might have changed. Hence, findings should be noted with caution; inference for cases in recent years and comparison with prevailing estimates among the elderly for different time periods are limited. Third, societal criminalization of suicidal behaviours might have affected respondents’ willingness to fully disclose their wish to die thoughts and provide accurate responses for other self-reported experiences. The current design prohibits causal inference of the noted associations, as the measurement of exposure variables and the condition (the wish to die) was conducted at the same point in time. However, the inclusion of critical demographic, social, economic and behavioural factors associated with the wish to die as cited in previous studies warranted their examination. A prospective follow-up study or longitudinal study on the subject themes would provide more accurate information and may assist with a better understanding of the phenomena over time rather than the cross-sectional design employed in the present study

## 5. Conclusions

The present study has demonstrated that psychosocial (hopelessness, depression, social connectedness), socio-demographic (i.e., age, sex, education, income and employment), anthropometric (BMI), and other factors correlate with the wish to die among persons aged 65 years and above in Ghana. The findings highlight the need for the timely identification of those at risk for appropriate intervention in order to promote their wish for longer life expectancy. Thus, to reduce the risk of the wish to die among the elderly in Ghana, it is recommended that indigent elderly be given the needed geriatric mental healthcare and palliative care to boost their desire to live longer. 

## Figures and Tables

**Table 1:** Statistics on factors associated with the wish to die in Ghana.

	Weighted Distribution				
	Wish to Die				
	No	Yes	Total		
	*n* = 1147	*n* = 1000	*n* = 2147		
	100%	100%	100%		
**Predictors**				**χ^2^**	***p*-Values**
Age				38.42	<0.001
65–74	60.8	58.9	59.9		
75–84	27.4	30.0	28.6		
85+	11.8	11.0	11.5		
Sex				9.85	0.002
Male	47.1	45.6	46.4		
Female	52.9	54.4	53.6		
Residence				26.42	<0.001
Urban	39.1	38.7	38.9		
Rural	60.9	61.3	61.1		
Marital Status				24.39	<0.001
Married	44.8	46.8	45.7		
Not married	55.2	53.2	54.3		
Education				91.44	<0.001
Not Educated	31.2	39.3	35.0		
Primary	26.9	34.5	30.4		
Secondary	37.2	23.7	30.9		
Tertiary	4.7	2.5	3.7		
Ethnicity				57.96	<0.001
Akan	53.1	41.5	47.7		
Ewe	7.2	7.3	7.3		
Ga-Adangbe	10.4	9.6	10.0		
Gruma	5.1	3.7	4.4		
Grusi	1.0	0.7	0.8		
Guan	1.2	1.7	1.4		
Mande-Busanga	1.7	1.4	1.5		
Mole-Dagbani	2.4	2.9	2.6		
Other	18.0	31.2	24.2		
Religion				19.27	<0.002
None	4.0	6.8	5.30		
Christianity	69.2	62.0	65.9		
Islam	15.0	16.8	15.8		
Traditional	10.7	13.7	12.1		
Other	0.6	0.6	0.6		
Refused	0.4	0.1	0.3		
Physically Challenged				52.77	<0.001
Yes	30.1	16.6	23.8		
No	69.9	83.4	76.2		
Tobacco Use				9.28	0.002
Yes	26.2	26.9	26.5		
No	73.8	73.1	773.5		
Alcohol Consumption				8.9	0.003
Yes	52.6	57.9	55.1		
No	47.4	42.1	44.9		
Employment				4.56	0.033
Yes	55.4	52.4	54.0		
No	44.6	47.6	46.0		
Depression				15.40	<0.001
Yes	2.7	2.6	2.7		
No	97.3	97.4	97.3		
Asthma				0.24	0.624
Yes	6.0	3.5	4.8		
No	94.0	96.5	95.2		
Physically active				19.38	<0.001
Yes	58.0	67.3	62.3		
No	42.0	32.7	37.7		
Diabetes				9.43	0.002
Yes	4.5	3.6	4.1		
No	95.5	96.4	95.9		
Hypertension				23.07	< 0.001
Yes	19.6	11.9	16.0		
No	80.4	88.1	84.0		
Arthritis				4.18	0.041
Yes	19.4	16.4	18.0		
No	80.6	83.6	82.0		
Stroke				6.10	0.013
Yes	3.6	4.3	3.9		
No	96.4	95.7	96.1		
Hearing Impairment				0.43	0.51
Yes	2.7	2.5	2.6		
No	97.3	97.5	97.4		
Vision Impairment				40.52	<0.001
Yes	9.8	19.5	14.3		
No	90.2	80.5	85.7		
Body Mass Index				22.64	<0.001
Underweight	16.7	16.0	16.4		
Normal	57.1	63.3	60.0		
Overweight	17.7	14.2	16.1		
Obese	8.5	6.5	7.5		
Injuries				19.14	<0.001
Yes	31.9	47.4	39.1		
No	68.1	52.6	60.9		
Wealth Quintile				100.37	<0.001
Poorest	15.0	29.1	21.6		
Poor	19.3	24.0	21.5		
Middle	21.6	20.1	20.9		
Richer	22.6	14.5	18.8		
Richest	21.5	12.3	17.2		
Hopelessness				235.88	<0.001
Yes	55.9	86.5	70.1		
No	44.1	13.6	29.9		
Religious Activities				4.10	0.042
Low	39.5	43.1	41.2		
High	60.5	56.9	58.8		
Having Friends Over				4.15	0.042
Low	24.8	24.5	24.6		
High	75.2	75.5	75.4		
Social Meetings Outside Home				9.49	0.002
Low	37.0	36.2	36.6		
High	63.0	63.8	63.4		

Source: Computed from SAGE, Wave 1 (Ghana), 2007–2008; *n* denotes the number of observations; *p*-values denote t test statistical significance; χ^2^ denotes chi-square estimates; *p* < 0.05, *p* < 0.01, *p* < 0.001, statistical significance.

**Table 2:** Multivariate logit model on factors predicting the wish to die.

	Wish to Die (*n* = 2147)	
	a^1^ = No, Yes	
	Model A	
Predictors	AOR	95% CI
Age		
65–74	a^1^	a^1^
75–84	** 1.05	1.02–1.16
85+	*** 1.48	1.22–1.94
Sex		
Male	a^1^	a^1^
Female	** 0.48	0.30–0.95
Residence		
Urban	a^1^	a^1^
Rural	** 0.64	0.50–0.81
Marital Status		
Married	a^1^	a^1^
Not married	*** 1.63	1.49–1.81
Education		
Not Educated	a^1^	a^1^
Primary	** 0.69	0.50–0.98
Secondary	** 0.57	0.44–0.76
Tertiary	*** 0.43	0.23–0.72
Ethnicity		
Akan	a^1^	a^1^
Ewe	0.96	0.59–1.44
Ga-Adangbe	1.20	0.85–1.68
Gruma	0.74	0.55–1.17
Grusi	2.17	0.69–4.21
Guan	1.82	0.81–3.16
Mande-Busanga	0.96	0.43–2.17
Mole-Dagbani	0.84	0.63–1.56
Other	*** 2.43	1.77–3.34
Religion		
None	a^1^	a^1^
Christianity	** 0.59	0.36–0.95
Islam	*** 0.36	0.20–0.63
Traditional	*** 0.31	0.18–0.53
Other	0.35	0.18–1.36
Refused	1.06	0.27–2.09
Physically Challenged		
Yes	a^1^	a^1^
No	*** 0.57	0.43–0.79
Tobacco Use		
No	a^1^	a^1^
Yes	*** 1.69	1.53–1.90
Alcohol Consumption		
No	a^1^	a^1^
Yes	** 0.1.83	1.66–2.06
Employment		
Yes	a^1^	a^1^
No	** 1.73	1.37–2.21
Depression		
No	a^1^	a^1^
Yes	*** 1.21	1.03–2.22
Physically active		
Yes	a^1^	a^1^
No	** 1.12	1.05–1.20
Diabetes		
No	a^1^	a^1^
Yes	** 1.07	1.01–1.43
Hypertension		
No	a^1^	a^1^
Yes	*** 1.49	1.36–1.66
Arthritis		
No	a^1^	a^1^
Yes	* 1.11	1.03–1.20
Stroke		
No	a^1^	a^1^
Yes	*** 1.28	1.10–1.86
Vision Impairment		
No	a^1^	a^1^
Yes	*** 3.05	2.25–4.16
Body Mass Index		
Normal	a^1^	a^1^
Underweight	* 1.28	1.03–1.41
Overweight	1.02	0.69–1.35
Obese	*** 1.33	1.03–1.54
Injuries		
Yes	a^1^	a^1^
No	* 0.84	0.71–0.93
Wealth Quintile		
Poorest	a^1^	a^1^
Poor	*** 0.84	0.77–0.92
Middle	*** 0.72	0.56–0.93
Richer	*** 0.63	0.44–0.89
Richest	** 0.49	0.33–0.72
Hopelessness		
No	a^1^	a^1^
Yes	*** 2.15	2.11–2.20
Attending Religious Activities		
High	a^1^	a^1^
Low	* 1.05	1.01–1.21
Having Friends Over		
High	a^1^	a^1^
Low	* 1.03	1.01–1.07
Social Meetings Outside		
High	a^1^	a^1^
Low	** 1.30	1.04–1.63

Source: Computed from SAGE, Wave 1 (Ghana), 2007–2008; Note: AOR denotes adjusted odds ratio; *n* is the number of observations; CI denotes confidence interval; a^1^ represents reference level; * *p* < 0.05, ** *p* < 0.01, *** *p* < 0.001, statistical significance.

## Data Availability

The data used for the study is available at: https://apps.who.int/healthinfo/systems/surveydata/index.php/catalog/6.

## Abbreviations

BMI	Body Mass Index
AOR	Adjusted Odds Ratio
AIC	Akaike Information Criterion
SAGE	World Health Organisation Study Global Ageing and Adult Health Survey
SSA	Sub-Saharan Africa
WHO	World Health Organisation
